# Developing a Web-Based Comic for Newly Diagnosed Women With Breast Cancer: An Action Research Approach

**DOI:** 10.2196/10716

**Published:** 2019-02-04

**Authors:** Tzu-I Lee, Shuh-Jen Sheu, Hsueh-Chin Chang, Yu-Ting Hung, Ling-Ming Tseng, Shin-Shang Chou, Te-Hsin Liang, Hui-Ju Liu, Hui-Ling Lu, Mei-Chun Chen, Ying-Chun Liu, Chi-Shan Tsai, Jui-Chiung Sun

**Affiliations:** 1 School of Nursing National Yang-Ming University Taipei Taiwan; 2 Institute of Community Health Care School of Nursing National Yang-Ming University Taipei Taiwan; 3 Public Health Center of Taoyuan District Department of Public Health Taoyuan City Government Taoyuan Taiwan; 4 Comprehensive Breast Health Center & Division of General Surgery Department of Surgery Taipei Veterans General Hospital Taipei Taiwan; 5 Department of Surgery School of Medicine National Yang-Ming University Taipei Taiwan; 6 Department of Nursing Taipei Veterans General Hospital Taipei Taiwan; 7 Department of Statistis and Information Science Fu Jen Catholic University New Taipei City Taiwan; 8 New York University New York, NY United States; 9 Health Education Taiwan Tanabe Seiyaku Co, Ltd Taipei Taiwan; 10 Department of Nursing National Taiwan University Hospital Taipei Taiwan; 11 School of Nursing National Defense Medical Center Taipei Taiwan; 12 Wan Fang Hospital Taipei Taiwan; 13 Department of Nursing Chang Gung University of Science and Technology Taoyuan Taiwan; 14 Department of Obstetrics and Gynecology Linkou Chang Gung Memorial Hospital Taoyuan Taiwan

**Keywords:** action research, breast cancer, comic, narrative

## Abstract

**Background:**

Personal narratives have been seen as a useful way of communicating about cancer treatment options and providing recovery information. Many printed versions of such material are available, including comics that explore the individual memories of patients who have gone through cancer treatment. These studies have been used to orientate patients, patients’ relatives, and physicians. However, only a few Web-based comics have been specifically designed for patients with breast cancer and used as aids to decision making.

**Objective:**

We aimed to describe the developmental process of creating an animated comic as a Web-based surgery decision-making tool; the comic was aimed at illustrating the feelings, thoughts, and meanings when a patient suffers from breast cancer. This was done by recounting the symptoms, diagnostic process, treatments, and treatment effects of such women from the diagnosis stage onward.

**Methods:**

Using cycles of planning, action, evaluation, and reflection, which involved collaborative work, action research was conducted to develop a Web-based animated comic. The stages of action research consisted of (1) semistructured and in-depth interviews to collect experiences of women with breast cancer; (2) construction of an animated comic by editors, graphics designers, dubbers, and information technology engineers; (3) redrawing of pictures of the comic after gathering feedback from a breast surgeon; and (4) evaluation of the Web-based animated comic using 6 patient focus groups.

**Results:**

The comic was produced and showcased on the website “The Network of Making-decision Aids for Breast Cancer Surgery”; the comic was accompanied by soft music and audio explanations. The comic functions as a personal statement that describes experiencing breast cancer. The animated comic consists of 8 chapters, based on the 8 themes deducted from the findings obtained during the analysis of relevant interviews. The 8 chapters include (1) the appearance of a lump; (2) confirmation by medical diagnosis; (3) the uncertainty of waiting (4) fear of life-threatening disease; (5) choosing life over despair; (6) being brave and deciding to undergo treatment; (7) choosing the type of surgery; and (8) being reborn.

**Conclusions:**

Using action research, this study illustrated that the comic that sheds light on issues of feelings, emotions, and thoughts that are present when a woman is diagnosed with breast cancer and provides a communication medium to explain the steps in the process. Meanwhile, it implies that hope will be able to overcome the challenges that will be faced. Within the Web-based decision aid for patients with breast cancer, the animated comic acts as an information resource and is aimed at patients’ understanding of impacts of emotions arising when suffering from breast cancer. It is potentially applicable as a therapeutic tool that facilitates self-reflection and self-healing among newly diagnosed patients with breast cancer.

## Introduction

Over the past few years, the number of breast cancer cases has been consistently increasing worldwide [1]. Over the same period, breast cancer has been ranked as the primary cause of cancer-related death among women in Taiwan [2]. Most patients with early-stage breast cancer are presented with lumpectomy or mastectomy as the recommended treatment [3]. Generally, after being diagnosed with breast cancer, patients must make a decision regarding the type of surgery they would prefer to undergo. However, even without having decided on the type of surgery, most hospitals in Taiwan offer their patients a surgery date, which normally ranges from 1-2 weeks in the future. With such a constrained time window owing to the treatment process, patients are usually asked to make a quick decision regarding the type of surgery they prefer. In Taiwan, most breast surgeons and oncologists still focus mainly on the biomedical aspects of treatment, paying much less attention (perhaps, unwillingly) to the emotional impact of this condition on patients and their relatives [4].

Psychologists have shown that emotions do, indeed, play an important role in the patient decision-making process regarding the type of treatment they want to undergo when treating their cancer [5]. From the moment they are diagnosed with the condition, patients begin to experience different levels of emotional disturbance [5] such as shock, anxiety, uncertainty, ambiguity, sadness, anger, guilt, fear of death, fear of suffering, and depression [5-8]. For most women, breast cancer becomes an overwhelming emotional experience [7]. The breast “cancer diagnosis” can provoke reactions of the negative emotions mentioned above and inflate a range of emotional experiences [5-8]. This wide range of emotions and psychological episodes may end up submerging a patient and her relatives in a state of intense uncertainty [8]. Briefly, a woman who gets diagnosed with breast cancer might be daunted by the uncertainties associated with the prognosis and treatment [8,9] and by considerations of herself, her relationships, and her life [10]. Research has shown that breast cancer is often very stressful for women from the very moment of diagnosis and all the way through surgery, including the period when they are waiting for diagnostic results [7,11,12]. In addition, the way these women are diagnosed can directly cause an increase in their stress level, which strengthens negative emotions [13]. Moreover, time constraints in the clinical setting in addition to certain aspects of the Chinese social culture may contribute to aggravating the situation. For instance, the fact those patients are not too willing to openly discuss cancer-related feelings [14]. Therefore, this makes them feel alone and not understood [15]. In this context, the specific needs of given individual’s psychological issues might not be properly identified and addressed [15,16]. Studies have found that being diagnosed with cancer may affect a patient’s cognitive capability by temporarily reducing comprehension and the ability to process information [17,18]. The studies cited above provide evidence of the impact that negative emotions can have during the diagnosis period. This indicates that assisting women during this stage should be able to contribute to more informed and conscious decision making about the type of surgery and type of treatment better suited for them.

There are several alternatives ways to provide emotional support to patients with breast cancer in a clinical setting; these include support groups, individual counseling, and others. These kinds of arrangements often seek to manifest narratives naturally. Personal narratives are often used in the realms of health and medicine to make up for lack of experience, to help the patients’ interpretation of their health status, and to embody such status [19]. Through narratives, a person undergoing a physical illness may be able to make sense of or revise his or her experiences during the illness [19]. This is because, when narratives are used as a medium, it helps the environment surrounding a person to be understood, which, in turn, informs a person about his or her concept of himself or herself [20]. This allows patients to make better sense of events and attempt to act in accordance with the meanings they have gathered [21]. Once it is understood that narratives construct a story, research has been able to show that cancer survivors’ stories not only have a positive influence on both the listeners’ and storytellers’ health [22-24] but it is also clear that they have a positive effect by reducing a reader’s or a listener’s feelings of fear and isolation during his or her illness [25]. This shows that patient stories seem to be more authentic, credible, and appealing compared with the mere documentation of facts [26].

Comics are a type of narrative that integrates sequential visual imagery and text [27]. Comics can convey affective and aesthetic sensibilities that have an impact on the affective processes and responses of readers [28,29]. They can also make use of a set of visual narrative interpretation structures that allow them to tell complex and emotionally rich stories, while conveying, at the same time, information without an individual having to read any complex text [30]. Comics are uniquely suited to represent vivid and valid embodied illness experiences [30]. Several prominent cancer-related comic narratives from the patients’ and family perspectives have been published such as Marisa Acocella’s “Marchetto’s autobiographical Cancer Vixen” (breast cancer) [31], Brain Fies’s “Mom’s Cancer” (lung cancer) [32], and Stan Mack’s “Janet and Me” (breast cancer) [33]. These comics represent individual experiences that are relevant to a specific illness or type of cancer and include the medical and diagnostic approaches to the disease in question [34]. In addition, they include the dialogical use of images by purposely adjusting hue, tone, saturation, and colors conjointly in parallel with the written language. These processes are used to deploy a given narrative and, as a result, the comics can catch and juxtapose multiple different layers of cancer-embodied experiences, especially those that are unable to be adequately and fully captured by narrative alone [31-33,35]; this can be done in the comics from various perspectives, namely the patient, the patient’s family members, and the patient’s caregivers [31-33,35].

Emotions can influence humans’ decision making [36,37]. A few stories of the breast cancer journey have focused on women with breast cancer before undergoing surgery and decision making. Where they exist, these stories do not necessarily include comprehensive descriptions or analysis of the psychological impacts of a breast cancer diagnosis. Furthermore, and obviously, illustrated comics with this discourse are even rarer. We believe that alleviating the potentially pessimist mindset of women with breast cancer is an important task during the decision-making process. Hence, the purpose of our creation of a comic story is to serve as a psychosocial support tool, which is aimed at helping newly diagnosed patients with breast cancer so that the possibility of emotional distress is reduced during the decision-making period for treatment.

## Methods

### Design

An action research (AR) design was utilized to generate the comic through engagement with women who have had real experiences with breast cancer. AR implies “research with people” instead of “research on people” [38]; it works in a mutually cooperative way linking researchers and participants to develop a project [38,39]. AR has various forms, different emphases, and several key principles, all of which have been presented in the literature [40,41]. AR is a diversified, reflexive, and cyclical research methodology that typically uses several different methods to collect and analyze data that are regardless of the data’s various forms [38,40,41]. AR is characterized by a cyclical and continuous interleaving of planning, action, evaluation, and reflection [39]. AR does not seem to have been applied to the design of Web-based decision-making aids for patients with breast cancer before. With thorough analysis and team discussions, we concluded that applying AR, in combination with several different data collection methods, was the most suitable and flexible way of conducting this study. It allows more brainstorming and creative (innovative) thinking and performance emergence. This mixed-method gives us deeper understanding, which, in turn, allows us to build a fuller picture; this then could be translated into the creation of our proposed Web-based animated comic.

**Figure 1:**
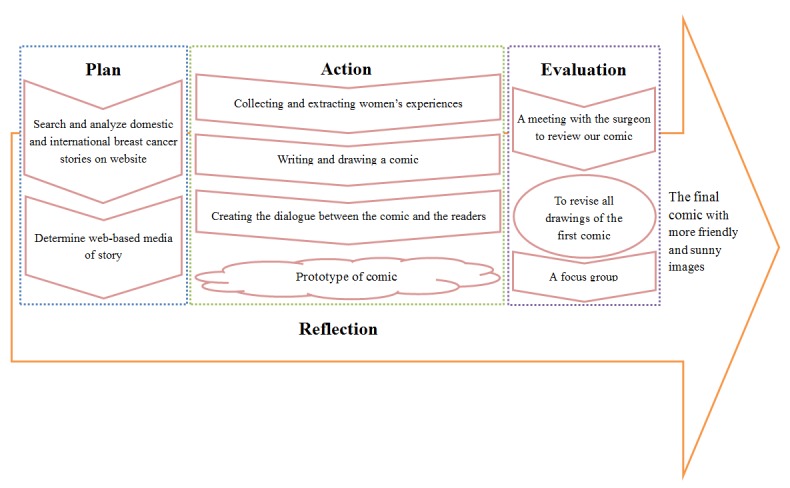
Research Process.

The cyclical process of our AR included the sequential development of our outline, drafting of a script, and related graphics (this needs to be easily understandable by any audience with a middle school level of education), recording of the script, and developing of animated graphics to produce an initial prototype of the Web-based comic (see Figure 1). Progress and the accuracy of the project were assessed using biweekly or weekly team meetings, during which adjustments were made where needed after reaching a consensus among all the team members. Our research team included a Principal Investigator (SJS), nursing students of different levels, information technology engineers, and computer graphics designers. This research was approved by the hospital’s Institutional Review Board, and informed consent was obtained from all research participants.

### Planning Phase

During the Planning Stage, the team defined and planed a Web-based multimedia module. This was designed by searching and analyzing Web-based personal stories of patients with breast cancer. We moved forward with the designing of a Web narrative-comic structure by applying visual aesthetics and the healing concept. Working on the premise that an animated comic as a breast cancer decision-making aid needed most importantly to provide accurate, empathetic, and easy-to-understand emotional information for patients with newly diagnosed breast cancer, we emphasized both the visual and psychological effects of the comic.

### Action Phase

During this stage, we proceeded with the design of the animated comic, which was constructed on the basis of the results obtained from the detailed analysis of the interviews with patients with breast cancer. Particular attention was paid to the media aspects and content aspects of the comic, with the leading investigator and the rest of the team focusing on these from the outset of the project. To allow the interviews with patients guide the development of the final comic, this phase followed the following 3 steps.

#### Step 1: Collecting and Extracting Women’s Experiences

We used purposive sampling to recruit 11 patients with breast cancer from a hospital in Taipei, Taiwan, between September 2010 and July 2011 (average age, 49.5 years; range 41-60 years). Among these patients, 5 had undergone mastectomy, 5 had undergone lumpectomy, and 1 had undergone a mastectomy followed by immediate breast reconstruction. We conducted semistructured individual interviews, using prompt, reflective, and open-ended questions in a conversational style to facilitate the participants’ conversions about a range of topics associated with their experiences with breast cancer. All interviews were transcribed verbatim. The data collection was based on the principles of saturation. We used the Objective and Systematic Approach of Miles and Huberman for the content analysis of the transcripts [42]. The text of each interview was cataloged and analyzed. Furthermore, the frequency, order, and intensity of the occurrence of words, phrases, or sentences related to themes that represented specific concepts were enumerated for each participant [42].

Findings of our analysis of the interviews indicated that women with breast cancer were dealing with conflicting emotions and thoughts that resulted from being diagnosed with breast cancer. A simple thematic analysis of the interviews’ data revealed 8 themes. These thematic perspectives were closely associated and appeared to affect the ways in which these women described their experiences of being diagnosed with breast cancer. In addition, these themes were related to the physical, emotional, and social perspectives of patients, especially since the diagnostic period through presurgery. The 8 emerging themes included (1) the appearance of a lump; (2) confirmation by medical diagnosis; (3) the uncertainty of waiting; (4) fear of a life-threatening disease; (5) choosing life over despair; (6) being brave and deciding to undergo treatment; (7) choosing the type of surgery; and (8) being reborn.

#### Step 2: Writing and Drawing the Comic

##### Writing the Comic

The collection of individual interviews provided us with actual dialogue and character descriptions that were then incorporated into one storyline of the comic to ensure that it would be relevant to our targeted audience and relevant situations. Upon comparing it with the narratives of all participants, 1 participant’s story resonated especially with us, and it became the main plotline for our comic. This woman had undertaken many roles, tasks, and responsibilities during her life, including maintaining her marriage, raising a child, and so on; her story and life experiences conveyed a wide array of feelings worth incorporating into our comic. Based on the purpose of our research and our targeted audience, we thought one storyline was enough in this study because aspects of multiculture and different races are not significantly present in the Taiwanese population. We employed the 8 themes resulting from the interview data as individual chapters of the comic’s plot. The narrative of each chapter is the content extracted from these interviews after they were correctly modified to reflect typical scenarios that result from the experience of being diagnosed with breast cancer.

**Figure 2:**
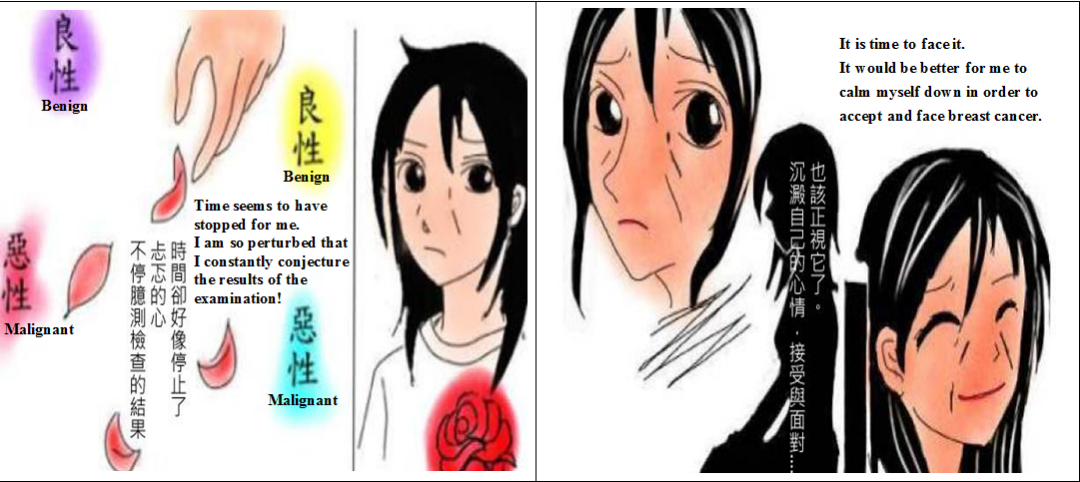
Sample drawings from the initial comic.

##### Drawing the Comic

After a thorough discussion and consultations among team members, we agreed on the inclusion of 32 slides in the form of webpages, 4 slides per theme to make up our proposed animated comic strip. While the team discussed the context of these interviews and the comic plot, 1 comic illustrator was asked to sit in during meetings to listen to the discussion or even take part where appropriate. The illustrator could draw during meetings or produce the images after reflection at a later time. The pictures of the comic are simple hand drawings influenced by manga and stresses on the facial expressions of the character within which the emotions are expressed and perceived. From descriptions of the team members, the illustrator captured some facial expressions and actions of the character in this comic from patients whom the team members had met and cared for previously or further researched patient blogs and self-help sites, as well as academic papers. The team then held a series of iterative discussions to determine the final list of comic images, which best exemplified the comic scenario and were sketched and redrawn several times before they were agreed on. Representative quotes are shown in the comics to point out participants’ cancer-related emotions and thoughts and, thus, provide a more holistic context.

To enhance a better user’s experiences and more comprehensive involvement in short viewing time, audio explanations and soft music tracks were embedded within the comic. Overall, all pictures have been drawn so that they purposefully follow a simple color-matching structure, which, combined with the oral and textual narrative, embodies the healing and calming purpose of the comic (see Figure 2).

#### Step 3: Creating a Dialogue Between the Comic and its Readers

We added 6 questions from the Chinese version of the *Mishel Uncertainty in Illness Scale* [43] and 8 questions from the *Mini-Mental Adjustment to Cancer Scale—Chinese Version* [44] to the slide at the end of each theme to provide the readers with an opportunity to confront and understand their cancer-related feelings and thoughts. Following these questions, an additional blank column was provided on the same slide to allow readers writing their own feelings and thoughts.

### Evaluation Phase

The evaluation of developing comic was conducted by interviews with a breast cancer surgeon, focus groups meetings with former patients with breast cancer, and an internal assessment by regular team meetings.

#### The Surgeon’s Comment

Once the animated comic was developed, we convened a meeting with a renowned breast surgeon in Taipei (LMT) to have him assess our work and provide some feedback. The surgeon gave our work an overall positive review; however, he expressed some concerns regarding the drawings, specifically regarding the appearance of the character in the comic. He suggested that she appeared to be too sad and transmit sorrow to the comic’s audience, especially to patients with newly diagnosed breast cancer. He pointed out that, based on his experience dealing with these kinds of patients, they might have a far more pessimist mindset than health providers can see and expect in such a situation. Moreover, he indicated that such women might not be able to afford any extra negative emotional suffering; therefore, he deemed it unnecessary to expose this group to a sad and depressive visual stimulation through the comic-related images.

#### Response to the Surgeon’s Comments

Considering the surgeon’s comments, we invited and tasked another different graphics designer to review and redraw of our first comic. As this graphics designer reflected on the explanations of the comic storyline from the initial design, the pictures of the initial comic, and his drawing experience, he was simultaneously acting as a viewer and (re)positioning himself as an illustrator. Furthermore, he was deeply considering viewers in the shoes of newly diagnosed breast cancer women. Upon agreement of the team, he used the same comic storyline but expertly laid down a different drawing style from the first comic through using images, color, symbolism, and spatial arrangement of objects. Still, facial expressions and actions of the character in this new-version comic kept the rigorous drawing process of the first version comic. Therefore, the second-version comic was made more visually friendly, thus conveying a more optimistic feeling.

#### Feedback From the Focus Groups

Upon completion of the second-version comic, we formed a focus group with the aim of helping us to evaluate the experience and impact of our work on real users once it had been launched to the general public [42]. We believed that a focus group was the best way to conduct such an evaluation, especially because it offered participants an opportunity to openly and freely express their personal thoughts and emotions [42]. This, in turn, would allow us to generate and collect a rich dataset [42]. The goals of the focus groups were to obtain a broad spectrum of feedback regarding the framework of the comic strips, as well as to gather ideas for promoting the comic, and receive suggestions on ways to improving it. We conducted 6 focus group meetings, which comprised 7 breast cancer survivors (average age, 60.4 years; range 51-72 years), from January 2015 to March 2015. Overall, 6 of these participants had been diagnosed with stage I cancer, while 1 had been diagnosed with stage II. They all had undergone surgery between 1993 and 2014. Regarding this new comic, we did not receive any suggestions of a modified form from these focus group members but got some positive appraisal of this comic from them.

The focus group members were enthusiastic after viewing the 2 versions of the comic during focus group meetings. Their opinions of both versions of the comic were generally positive. Although they preferred the images of the initial comic, they were satisfied with the second version as well and understood why we had adopted the second-version comic based on the recommendation of the breast surgeon. In addition, they positively valued the plot and emotion descriptions within the comic, which they found easy to follow and understand. Furthermore, they indicated that they felt very touched and empathetic after watching the animated comic because the images from the comic were able to truly represent their very own feelings and experiences when suffering from breast cancer.

...This was a road I have walked through... The emotional information was complete... It was like a very detailed description of my journey.

C2, 164

I thought this comic was very vivid and realistic to me describing how I felt. And, it was my spiritual journey...

E2, 186

It felt real, it was very empathetic. As if I had just breast cancer at that time. Really, I did not know exactly what to do, and then it seemed like me, when I was afraid that others knew about my disease and I almost did not dare to ask what was going on with my breast. So, I could only discuss about my breast with my husband. In really, it would have been better to watch this kind of comic story on Internet. If I could have seen it, then I naturally would have felt calmer, right, and I also felt I was ok.

G6, 6

Moreover, they agreed that they would not have felt as alone during the course of their breast cancer after viewing this comic; this was because this comic would have made them feel less anxious, less depressed, and more cared about by others.

...she (the patient) had to face various ups and downs in her mind before making a decision about surgery. The effect of reading the comic would have made her feel that this is a general phenomenon and it is normal to experience emotional changes after receiving a breast cancer diagnosis. It can be said that the comic seems to provide empathy and mental support for her, and she could counsel herself with this because she was not alone.

A2, 193

It is actually quite healing due to the caring effect of this comic story.

A2, 198

Furthermore, the breast cancer survivors thought that character modeling was more real because it addressed their embodiment of the breast cancer journey. Finally, they suggested that the initial version of the comic ought to become a comic book and acts as another information resource for newly diagnosed patients with breast cancer in hospitals.

## Results

**Figure 3:**
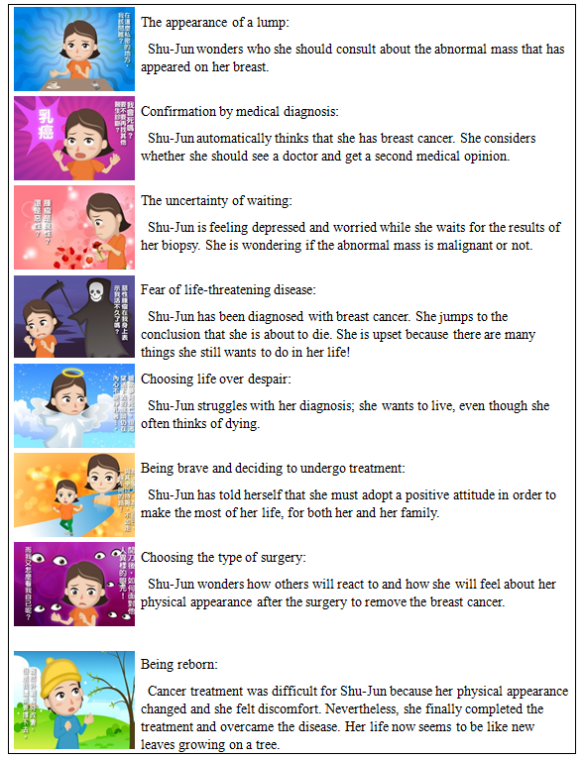
Examples of screenshots from the comic.

Based on the results obtained from analyzing patients’ interviews and, with the focus on “patient’s mindset while receiving breast cancer diagnosis,” the final product of our work was drawn by hand, digitally modified, and colored in Photoshop CC to be hosted on the website (Multimedia Appendix 1), where it was run for a relatively short time (8 minutes). The comic is accompanied by a female narration and soft background music. It consists of uncertainty, fear, and anxiety that are of intense emotional significance and which are conveyed through the flash-animated images. Named “Women’s Voice,” the story attempts to convey echoes of the personal words of patients with breast cancer. The comic combines narrative with strong visuals to present and communicate emotional information through the protagonist named Shu-Jun (a common Chinese women’s name). She autobiographically articulates her own life experiences with breast cancer, while simultaneously acknowledging the sufferings she had to endure along the way. Each theme’s full context is summarized in a screenshot (Figure 3).

## Discussion

### Principal Findings

“Cancer” can elicit negative images and stigmatizing responses from individuals associated with patients [45]. Furthermore, cancer-related emotional distress can affect both their thinking and information-processing capability of dealing with breast cancer [46] and their coping skills [47]. Even though there are many forms of mental support for women with breast cancer, mental support and emotional support seem to be difficult to define attending to the particular characteristics and culture of an individual. Comic serves as a medium for making trauma visible, and it does this by not only illustrating the fragmentation of such experienced memory [48] but also adds articulation to, otherwise incomplete, narratives, which often may be left unrepresented because they are hard to express or are too personal [48,49]. Compared with the form of plain text and printed material, which is helpful to certain extent, recent advances in digital content and information technologies inspired and allowed us to bravely create the available information in the form of Web-based animated comic story, which would be expected to be more accessible to wider groups of patients, caregivers, and relatives; this medium would also be appealing to the recipients [50]. Hence, the focus group members in this study highly recommended and appreciated viewing our Web-based animated comic to be as a consoling tool in future while they volunteer newly diagnosed patients with breast cancer in a hospital.

It is essential that care providers for patients with breast cancer let them feel that they are “not alone” and that they are “being supported” [51]. Compassion can make people become less self-absorbed and helps them to reach a better perspective regarding a problem [52]. Although one focus group member complained not to get used to the comic instead of a friend, the team hopes our comic, applying the drawing style, detailed expressions, and color aesthetics, would have relatable implications and allow reader-text intimacy; furthermore, it would give viewers suffering from breast cancer a sense of “we were in the same boat, we have also gone through the experience of having breast cancer, and we are not alone in this journey,” to induce feelings of compassion when a person faces breast cancer. From viewing the appraisal of focus group members, these women generally felt that the storyline was very realistic and then recalled that they experienced very touching feelings and deep empathy immediately after viewing our animated comic because our comic represents a character in a plot that is quite similar to their journey after receiving their cancer diagnosis.

Overall, focus group members appreciated the pictures and colors used in the comic and agreed that the use of bright colors added a description. We hope that our animated comic will become a healing story that exerts a positive influence on the wounds inflicted by breast cancer and that patients would be able to connect to other individuals through their experiences and thoughts after they have viewed this comic. However, this study is at the initial stage of developing the comic, therefore we cannot be sure whether this approach of reading the comic, the content of the comic, or both of them might be more meaningful for the field of mental support for Taiwanese women with newly diagnosed breast cancer. Furthermore, one single type or style of the comic content of this study is enough for the target of this study—the Taiwanese women population. Given that there are no multicultural and multiracial aspects to consider in Taiwan in the implementation of more than one type or style as would be the case in other multicultural or multiracial societies, we consider unnecessary to embark ourselves with the creation of more complex versions of this comic. Future evaluation studies of this comic approach will be used to assess the impact of comics as part of the wider health information and mental experience of women with breast cancer, as well as to evaluate this individual comic, including the narrative element and its visual component. Ideally, the results would benefit by repeating the project using empirical studies.

Interestingly, a preference difference existed between the breast cancer surgeon and focus group members with breast cancer regarding 2 different drawing styles of the same comic storyline. For the initial comic images, the surgeon suggested that the sad and depressed drawing style might contribute to making patients with breast cancer more miserable, more anxious, and more hopeless after reading this comic. However, focus group members thought these images were closer to their deeper insights of suffering breast cancer. They suggested that because facial expressions in this comic looked more like photographs and were more realistic than those in the second version and that they better represented the emotional conflicts and thoughts that they could not find the right words to express. Some researchers have pointed out that age, gender, cultural beliefs, and values have various influences on responses to the diagnosis and treatment of breast cancer [45,50] and, thus, might affect what is perceived when watching a comic of this nature [29]. This may explain the different senses of the 2 versions of the comic by the surgeon and patients. This kind issue of having different perceptions about organizing the Web-based comic construction also made the team to think more and overcome some challenges, such as aspects of different professional terminology, communication, information engineering, and art design, as well as how to comfort patients dealing with the effects of being diagnosed with breast cancer. Compared with conventional research designs, the AR cycles provided an opportunity to multiprofessionals working collaboratively to modify and confirm through each process repeatedly. This study may be regarded as a reference for describing how to develop a psychological and decision support tool for women newly diagnosed with breast cancer by considering their own corresponding culture and the region where they live.

### Conclusion

In this study, AR was used as the most appropriate methodological approach to understand the emotion-related suffering of newly diagnosed breast cancer women, leading to the development of our Web-based animated comic. We attempted to generalize the content of this animated comic about breast cancer stories thematically and simply to facilitate the readers’ comprehension of the emotional information. We believe this animated comic has the potential to serve as a positive influence on those women who need further orientation about the decision process regarding their choice of cancer-related treatment; this is because it seems better suited to their situation and provides these women with a channel that should help alleviate possible negative cancer-related emotions. This research is valuable because it presents and shares a different process of creation for a relevant media tool and because it uses a new creative way to bring a sense of hope to patients newly diagnosed with breast cancer.

## Appendix

Multimedia Appendix 1 The screenshot of the animation on the website.

## Abbreviations

AR: action research
