# Supplementary figures and images for: Developing a Web-Based Comic for Newly Diagnosed Women With Breast Cancer: An Action Research Approach
Source: J Med Internet Res. 2019 Feb 4;21(2):e10716. doi: 10.2196/10716 (PMC6378550; doi:10.2196/10716)

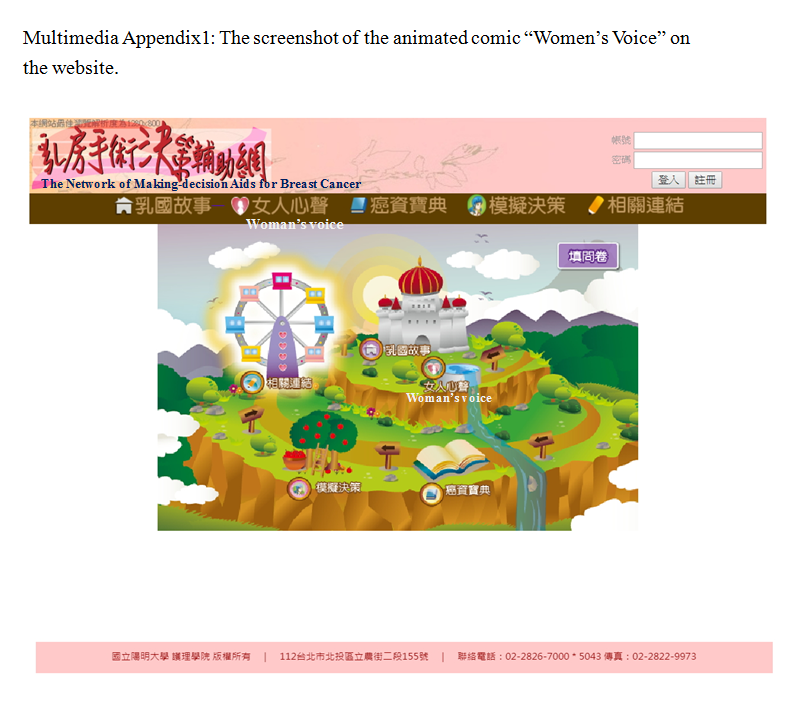

Supplement: Multimedia Appendix 1 [file jmir_v21i2e10716_app1.png]
